# Switching Behaviors of Graphene-Boron Nitride Nanotube Heterojunctions

**DOI:** 10.1038/srep12238

**Published:** 2015-07-20

**Authors:** Vyom Parashar, Corentin P. Durand, Boyi Hao, Rodrigo G. Amorim, Ravindra Pandey, Bishnu Tiwari, Dongyan Zhang, Yang Liu, An-Ping Li, Yoke Khin Yap

**Affiliations:** 1 Department of Physics, Michigan Technological University, 1400 Townsend Drive, Houghton, Michigan 49931, USA; 2 Center for Nanophase Materials Sciences, Oak Ridge National Laboratory, Oak Ridge, TN 37831-6487, USA; 3 Center for Integrated Nanotechnologies, Sandia National Laboratories, Albuquerque, NM 87185.

## Abstract

High electron mobility of graphene has enabled their application in high-frequency analogue devices but their gapless nature has hindered their use in digital switches. In contrast, the structural analogous, *h-*BN sheets and BN nanotubes (BNNTs) are wide band gap insulators. Here we show that the growth of electrically insulating BNNTs on graphene can enable the use of graphene as effective digital switches. These graphene-BNNT heterojunctions were characterized at room temperature by four-probe scanning tunneling microscopy (4-probe STM) under real-time monitoring of scanning electron microscopy (SEM). A switching ratio as high as 10^5^ at a turn-on voltage as low as 0.5 V were recorded. Simulation by density functional theory (DFT) suggests that mismatch of the density of states (DOS) is responsible for these novel switching behaviors.

Graphene is known for its high electron mobility and zero energy gap nature^1^,^2^. These have enabled their application in high-frequency analogue devices but hindering their use in digital switches^3^. Extensive efforts have been dedicated to generate band gap in graphene by using graphene nanoribbons^4^,^5^, graphene bilayer^6^,^7^, graphene on *hexagonal* phase boron nitride (graphene/*h-*BN)^8^, and applying strain on graphene/*h-*BN structures^9^. In contrast, *h-*BN sheets and BN nanotubes (BNNTs) are insulators^10^,^11^. The *h*-BN substrates are known to enhance electron mobility of graphene devices^12^,^13^. More recently, in-plane graphene/*h-*BN heterojunctions were also reported^14^,^15^,^16^. However, graphene digital switches have not been demonstrated in any of these graphene/*h-*BN heterostructures. Here we show that zero dimensional (0D) heterojunctions between BNNTs and graphene could switch current flows.

The graphene-BNNT heterojunctions are prepared by chemical vapor deposition (CVD)^17^,^18^, where BNNTs are grown on chemically exfoliated graphene without the use of catalysts (see Methods and Supplementary Fig. S1,S2). As shown in Fig. 1a,b, BNNTs were selectively grown on the graphene sheet and not on the surrounding oxidized Si substrates. The as-grown BNNTs point outward from the graphene surface at random angles. As shown in Fig. 1c,d, the self-assembled BNNTs are crystalline with tubular structure (Fig. S3), similar to those grown with the use of catalysts^11^. Raman spectroscopy (Fig. 1e, excitation laser wavelength = 325 nm), and electron energy loss spectroscopy (EELS, Supplementary Fig. S4) confirm the presence of BNNTs on graphene. The Raman spectra of the multilayered graphene before the BNNT growth are shown in the inset for comparison.

The as-grown graphene-BNNT heterojunctions were characterized for their electronic properties at room temperature by using a four-probe scanning tunneling microscopy (4-probe STM) system (see Methods)^19^. The 4-probe STM system allows us to observe the exact probing locations prior to current-voltage (*I-V*) measurements with the *in situ* SEM. Figure 2a shows an upright BNNT grown from the graphene surface. This BNNT was probed by a tungsten STM probe **1**, while another STM probe **2** was in contact with the graphene surface. The distance, *d*, between probe **1** and the vertical graphene-BNNT heterojunction, was controlled by changing the contact point on the BNNT.

For the case of d = 1.23 μm, we hardly detected any current flow across the graphene-BNNT heterojunction for a range of bias voltages, *V*_*b*_ (Fig. 2b,c) between −30 V and +30 V. The current noise level (i.e. “off” state) is about ~10^−11^A. As we changed *d* to 0.62 μm, we detected a significant higher level of current across the heterojunction (i.e. “on” state) with a threshold turn–on voltage, *V*_*on*_ ~0.5 V (inset). The current level detected at *d* = 0.10 μm is even higher, up to μA level (as limited by the preset 1 μA current limit), with *V*_*on*_ ~0.15 V (inset). These conducting behaviors with obvious “on” and “off” states are different from the reported insulating nature of BNNTs^18^,^20^. Such a switching behavior is also different from the metallic nature detected when both STM probes are in contact with the graphene surface (Fig. S7). These results suggest that graphene-BNNT heterojunctions could be used as electronic switches with an estimated switching ratio of 10^5^.

A full series of *I-V* curves were measured (supplementary Fig. S8). The detected current as a function of *d* at bias voltage V = 10 V and 15 V are extracted and plotted on two different scales in Fig. 2d,e, respectively. As shown, current across the graphene-BNNT heterojunctions decreased nonlinearly with the increase in the probe-heterojunction tunneling distance, *d.* Next, we investigated the gating effect on this vertical graphene-BNNT heterojunction. By keeping *V*_*b*_ ~10 V at *d* = 0.10 μm, the current flow across the heterojunction was monitored while applying a varying back gate voltage on the highly doped *p*-type Si 〈100〉 substrate (0.001–0.005 Ωcm, with a 500 nm thick Si oxide layer on top). As shown in Fig. 2f, a nearly constant current of 10^−7^A is detected. We believe that the absence of the gating effect is due to the shielding of the metallic graphene located between the gate oxide surface and the heterojunction. As the junction is small in diameter (~60 nm) and is potentially surrounded by additional graphene layers underneath, an electrostatic screening effect will be able to effectively shield the gating effect. Therefore, gating the junction should still be possible if a top gate is applied.

To understand these experimental observations, calculations were performed by employing Density Functional Theory (DFT)^21^,^22^ as implemented in the SIESTA code (see supplementary)^23^. The graphene-BNNT heterojunction was modeled in a supercell consisting of a graphene sheet and a BNNT placed perpendicular to the graphene sheet as shown in Fig. 3a. The calculated electron charge density for pristine graphene (upper row) and the graphene-BNNT heterojunction (lower row) is displayed in Fig. 3b. Here, a higher charge density is seen around the N atoms relative to that around the B atoms as expected. The calculated density of states (DOS) of the heterojunction together with the pristine graphene is shown in Fig. 3c. The shape of Dirac cones is well reproduced for the pristine graphene. On the other hand, DOS for the graphene-BNNT heterojunction is slightly shifted relative to that of the pristine graphene due to hybridization of C states with B/N states. Additional localized states are also predicted. There is an obvious mismatch of DOS at the nanoscale heterojunction between the graphene and the BNNT.

Next we calculated the electron transport properties of the heterojunctions using density functional theory together with the non-equilibrium Greens Function method (see supplementary). Fig. 3d shows the electronic transmittance at zero bias for both pristine graphene and graphene-BNNT heterojunction. The ballistic nature in pristine graphene is clearly predicted with a flat transmittance at the conducting range. For the heterojunction, the transmittance is suppressed, approaching zero at the Fermi level. The calculated *I-V* characteristics of pristine graphene and graphene-BNNT heterojunction are shown in Fig. 3e. The Ohmic-like *I-V* behavior for the pristine graphene is predicted affirming its metallic nature. On the other hand, the calculations predict very little, if any, electron transport through the graphene-BNNT heterojunction within a range of low bias voltages (i.e. ±0.1 V). At higher bias voltage, however, the linear *I-V* behavior is recovered with slightly higher resistance. The calculated switching properties of the heterojunction are in qualitative agreement with our experimental results.

We believe that the switching behaviors observed here is due to the DOS mismatch at the heterojunction^24^, as shown in Fig. 3c,d. Such a mismatch creates a potential barrier and prevents current flows through the heterojunction at low bias. At higher bias voltages, an enhanced electric field is expected as the potential gradient is confined at the nanoscale heterojunction, much like in electron field emission^25^. This enables electrons to either overcome or tunnel through the mismatch barrier, leading to current flow across the heterojunction. The dimension of such a heterojunction is small, approaching zero dimensional (0D) structures. Considering the most effective switching case at *d* = 0.10 μm, the effective dimension of the junctions is approximately ~120 nm in diameter.

We further examined the switching behavior of a horizontal graphene-BNNT heterojunction. Figure 4 shows the SEM image of a BNNT that is nearly horizontally extended from a graphene-BNNT heterojunction on to the surface of the oxidized Si substrate. As shown in Fig. 4a, an STM probe is in contact and pressing down the BNNT at a distance *d* = 0.64 μm away from the heterojunction. Another STM probe is in electrical contact with the graphene sheet (beyond the SEM imaging window). As shown in Fig. 4b,c, no significant current is detected at low *V*_*b*_ (“off” state) for both cases. At higher *V*_*b*_, the heterojunction turned “on” with thresholds *V*_*on*_ ~7 V and *V*_*on*_ ~4 V for *d* = 0.73 μm and 0.64 μm, respectively. Obviously, *V*_*on*_ for this horizontal heterojunction is higher than those discussed for a vertical heterojunction shown in Fig. 2. As schematically shown in Fig. 4d,e, bending BNNT will locally distort the tubular structure. Theoretically, such a radially deformed BNNT segment is known to change the band structure and reduce the band gap of BNNTs^26^. This additional DOS mismatch can increase the potential barrier, leading to higher *V*_*on*_. Such a DOS mismatch can only be artificially formed if local compression or severe bending occurs to the BNNT. It is unlikely to find such a deformation in as-grown BNNTs.

In conclusion, we have demonstrated the fabrication of the stable graphene-BNNT heterojunctions without the use of catalyst. A bias-dependent switching behavior has been observed at the heterojunctions. DOS mismatch between graphene and BNNT at the nanoscale heterojunction is suggested to prevent current flow at low bias voltages, and turn on the current flow at higher applied bias. The novel Graphene-BNNT heterojunctions would likely combine the advantage of the ballistic nature of electron mobility^27^ of single layer graphene and the switchable transport properties, when CVD synthesis of single-wall BNNTs is established in the field.

## Methods

### Synthesis of graphene-BNNT heterojuctions

**Figure 1:**
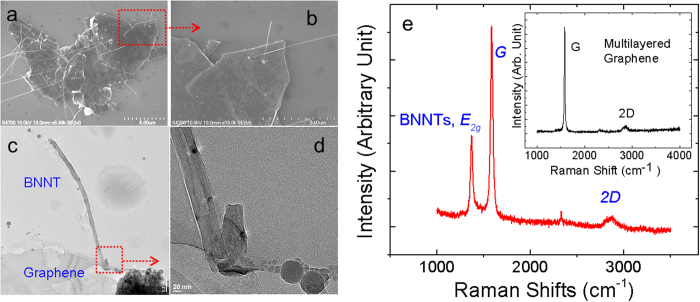
Structural Properties of graphene-BNNT heterojunctions. Microscopic images of graphene-BNNT heterojunctions obtained by (**a,b**) scanning electron microscopy (SEM) and (**c,d**) transmission electron microscopy (TEM). (**e**) Raman spectra obtained from as-grown graphene-BNNTs in comparison with the spectra from the multilayered graphene (inset). The diameters of these BNNTs are typically 20–80 nm.

Expandable graphite powders (Grade 3772, >98% carbon, Anthracite Industries, Inc., a subsidiary of Asbury Carbons, expansion ratio ~1:300) were heat shocked into multi-layered graphene at 1000 ^°^C in Argon ambient. These graphene sheets are then dispersed in tetrahydrofuran or isopropyl alcohol by sonication (10 minutes in a sonication bath). The suspended graphene was then coated on cleaned oxidized Si substrates and dried in hydrogen flow at 800 °C for 30 min. These graphene coated substrates are then used for the subsequence growth of BNNTs to form the heterojunctions. The growth of BNNT on graphene is based on our reported growth vapor trapping (GVT) chemical vapor deposition (CVD) method without the use of catalyst. These substrates were placed on the top of an alumina combustion boat in which B, MgO, and FeO precursors are loaded (total weight of 500 mg, with a mass ratio of 4:1:1). This setup was placed inside a closed-end quartz tube in the horizontal tube furnace with the graphene sheets facing upward. The precursors and substrates were then heated up to 1100–1200 °C with an ammonia flow of 200–350 sccm and kept for 30 min. Images of as-grown graphene-BNNTs heterojunctions are shown in Fig. S1 and Raman spectra of these heterojunctions are shown in Fig. S2. We believe that the growth of BNNTs is initiated by point defects, where dangling bonds serve as the nucleation sites. As some of the point defects are not round, the initially grown BNNTs became distorted (whitish dot in Fig. 1b, and Supplementary Fig. S3) but later converted into straight BNNTs as observed by transmission electron microscopy (TEM, Fig. 1c,d). These point defects are formed during the heat induced chemical expansion and exfoliation process. This interpretation is supported by the fact that BNNTs could not be grown on CVD graphene (Supplementary Fig. S5,S6). Apparently, the formation of graphene-BNNT junctions relies on the diameters of the point defects. We think that it is possible to form the graphene-BNNT heterojunction on mono- and multilayered graphene (at a reasonable range of graphene thickness of ~0.4–30 nm to match the diameter of typical single- and multi- walled BNNTs) as long as the point defects are found.

## Additional Information

**How to cite this article**: Parashar, V. *et al.* Switching Behaviors of Graphene-Boron Nitride Nanotube Heterojunctions. *Sci. Rep.* **5**, 12238; doi: 10.1038/srep12238 (2015).

## Supplementary Material

Supplementary Information

## Figures and Tables

**Figure 2:**
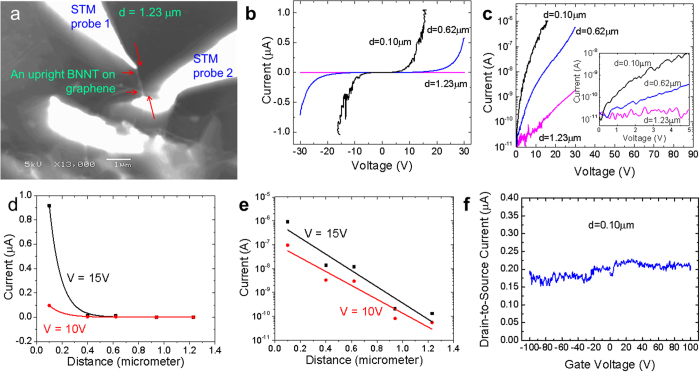
Distance dependent transport properties on a vertical heterojunction. (**a**) SEM image of a graphene-BNNT heterojunction as contacted across by two STM probes at conduction distance, *d* = 1.23 μm. (**b**,**c**) The corresponding current-voltage (*I-V*) characteristics at a series of *d*. (**d**,**e**) Linear and log scale of current flow across the heterojunction as a function of distance, *d* as extracted from Fig. S8. (**f**) The source-drain currents across the heterojunction as a function of back gate voltages.

**Figure 3:**
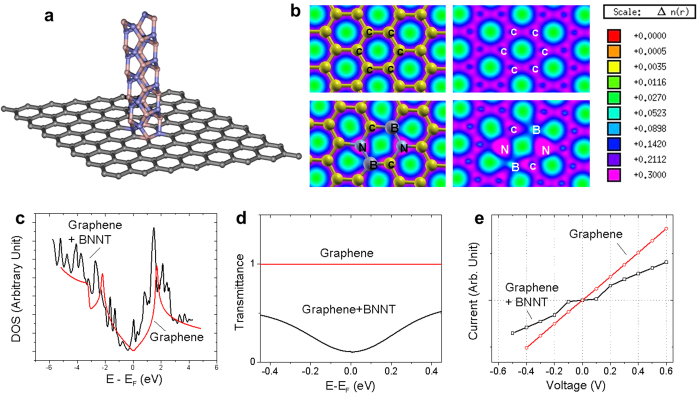
Theory. **(a)** Model of a vertical graphene-BNNT heterojunction. **(b)** Electron charge density of the pristine graphene (upper row) and the graphene-BNNT heterojunction (lower row). (**c**) Density of states (DOS), (**d**) the transmittance at zero bias, and (**e**) the current-voltage characteristics of the pristine graphene (upper row) and the graphene-BNNT heterojunction.

**Figure 4:**
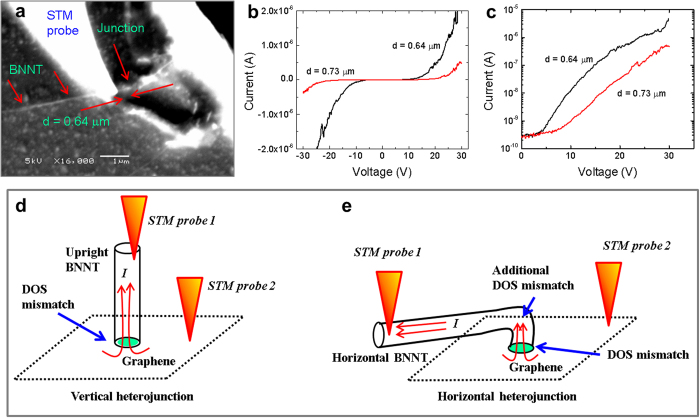
Distance dependent transport properties on a horizontal heterojunction and the schematic of DOS mismatch. SEM images of a horizontal graphene-BNNT heterojunction as contacted across by two STM probes (**a**) and the current-voltage (*I-V*) characteristics (**b,c**) at *d* = 0.73 μm, and *d* = 0.64 μm. Schematic drawing of a vertical heterojunction (**d**) and horizontal heterojunction (**e**) indicate the locations of DOS mismatch.
