# Supplementary material for: Switching Behaviors of Graphene-Boron Nitride Nanotube Heterojunctions
Source: Sci Rep. 2015 Jul 20;5:12238. doi: 10.1038/srep12238 (PMC4507443; doi:10.1038/srep12238)
Supplement: Supplementary Information [file srep12238-s1.doc]

**Supplementary Information**

Switching Behaviors of Graphene-Boron Nitride Nanotube Heterojunctions

*Vyom Parashar^1^, Corentin P. Durand ^2^,, Boyi Hao ^1^, Rodrigo G. Amorim ^1^, Ravindra Pandey ^1^, Bishnu Tiwari ^1^, Dongyan Zhang ^1^, Yang Liu ^3^, An-Ping Li ^2^, and Yoke Khin Yap^1, *^*

^1^Department of Physics, Michigan Technological University, 1400 Townsend Drive, Houghton, Michigan 49931, USA*.* ^2^Center for Nanophase Materials Sciences, Oak Ridge National Laboratory, Oak Ridge, TN 37831-6487, USA. ^3^Center for Integrated Nanotechnologies, Sandia National Laboratories, Albuquerque, NM 87185. ^*^e-mail: [ykyap@mtu.edu](mailto:ykyap@mtu.edu)

1. **Synthesis of graphene-BNNT heterojunctions**


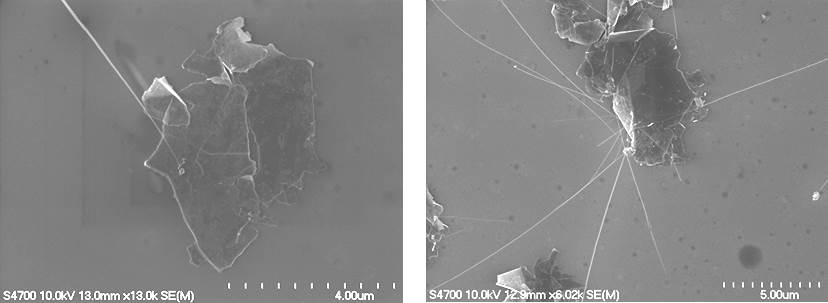


Figure S1: Additional SEM images of selectively grown graphene-BNNT heterojunctions.


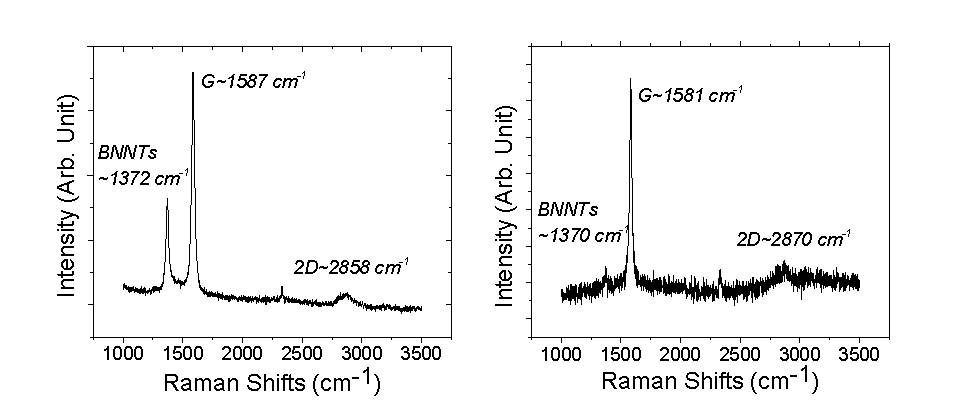


Figure S2: Raman spectra of graphene-BNNT heterojunctions with dense BNNTs (left) and sparse BNNTs (right). As shown the *G* and *2D* peak positions are varying with location while the BNNT peaks are about ~1370-1372 cm^-1^.

The Raman G peak position for graphene is ~1580 cm^-1^ but can be varied by strains. [^1^](#_ENREF_1) As shown in Figure S2, the G peak has significantly shifted when denser BNNTs are grown, potentially due to the strain induced by the formation of the heterojunctions. On the other hand, the Raman 2D peak position is very sensitive to the number of graphene layer as well as the wavelength of the excitation laser.[^2^](#_ENREF_2) For example, 2D peak as excited by HeNe (633nm) laser is ~2640 cm^-1^ and will shift to ~2825 cm^-1^ when excited by HeCd (325nm) laser.[^3^](#_ENREF_3) The 2D peak position can also be further shifted by strain.[^1^](#_ENREF_1)

1. **Transmission electron microscopy (TEM) and electron energy loss spectroscopy (EELS)**


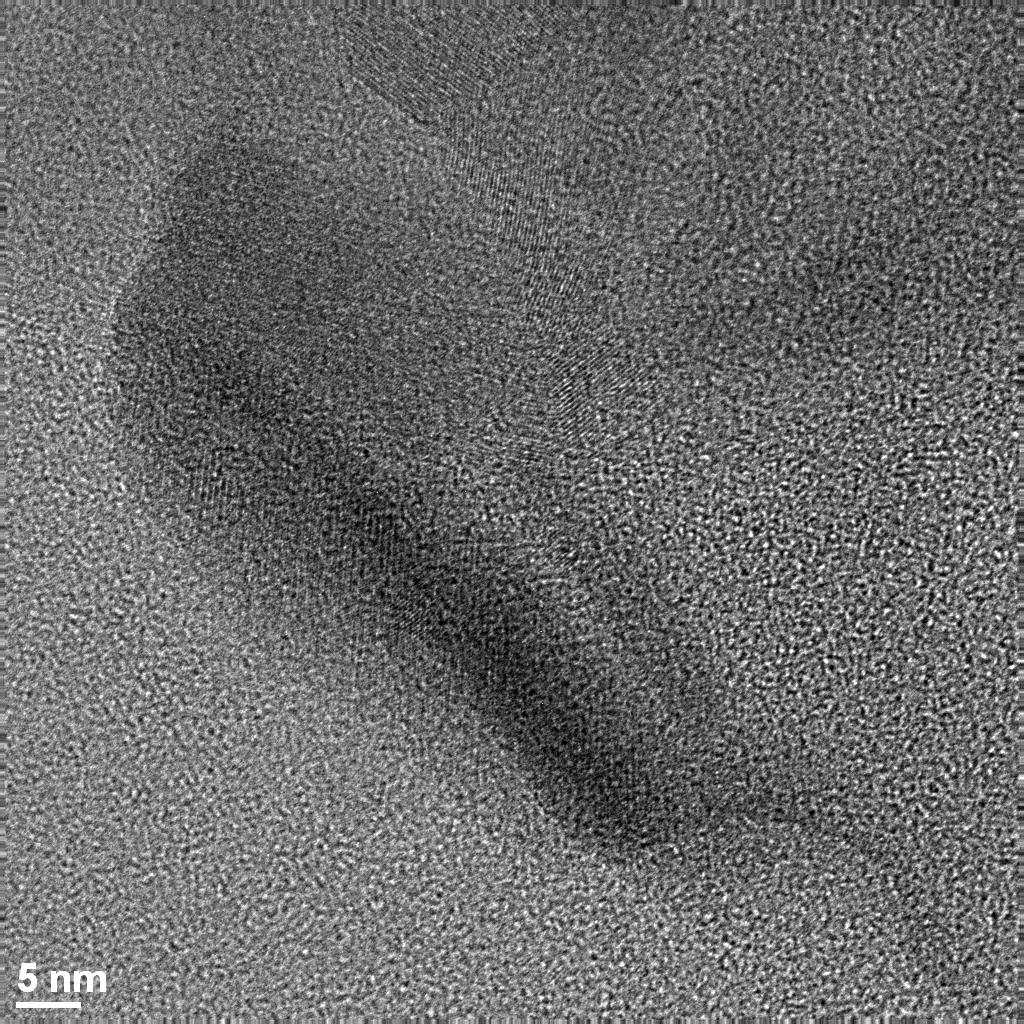

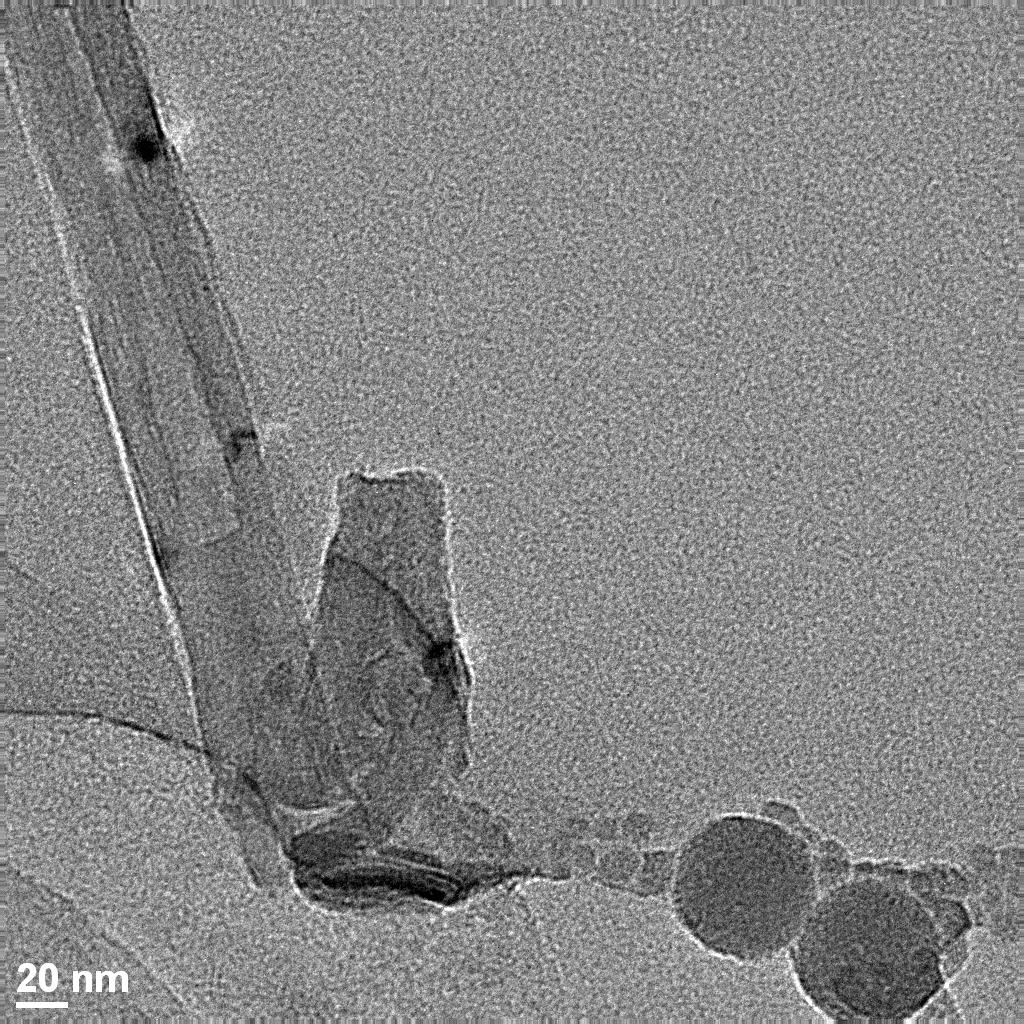

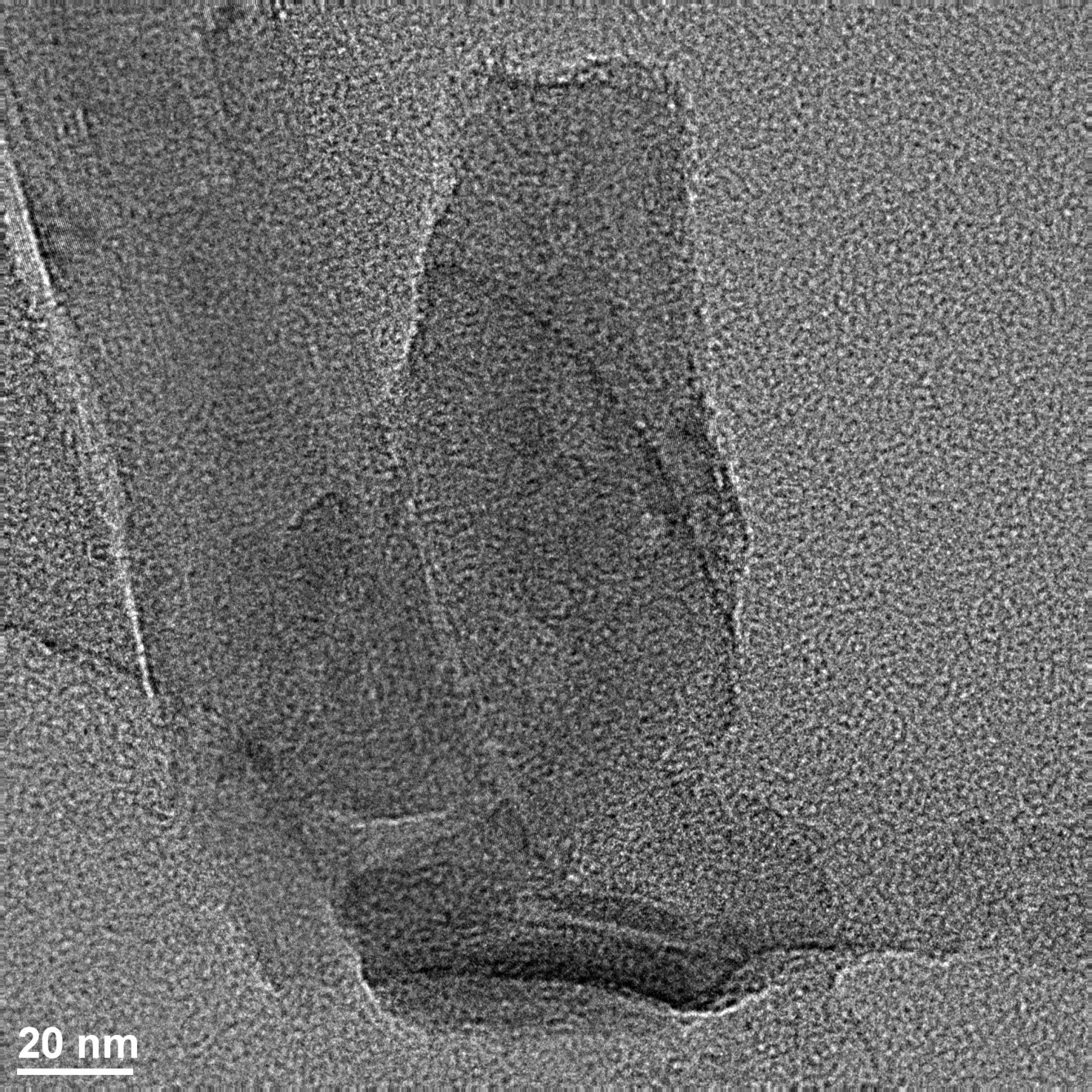


Figure S3: Lattice of randomly stacked hexagonal BN phase is detected at the graphene-BNNT junction.

TEM was employed to examine the structural properties at the graphene-BNNT heterojunction. A long BNNT and another broken BNNT are shown in Figure S3 (upper right). At the heterojunctions of graphene and these BNNTs, (lower right and left), highly crystalline hexagonal BN shells are detected in a randomly stacked manner. We interpret this as twisted / distorted tubular BN structure. Electron energy loss spectroscopy (EELS) was also employed to analyze the composites on the BNNTs and the graphene-BNNT heterojuction. As shown in Figure S4, the spectra for B, C, and N atoms are detected on the graphene-BNNT junction.


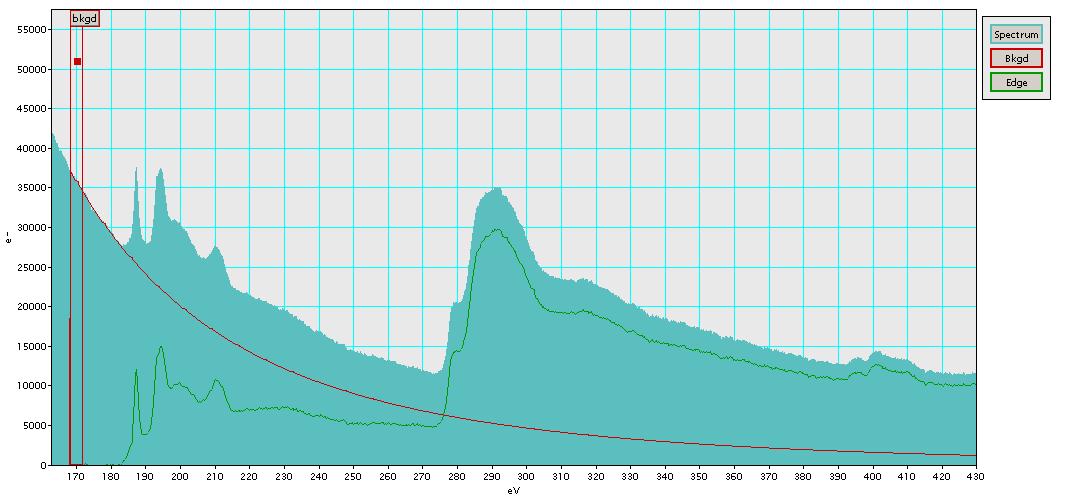


B-K

C-K

N-K

Figure S4: EELS spectra for B, C, N elements detected on the BNNT through the graphene layer.

1. **Attempts to grow BNNTs on CVD graphene**

We attempt to grow multiwalled graphene-BNNT heterojunctions using CVD graphene instead. These CVD graphene were grown on nickel (Ni) plates. These plates are cleaned by acetone, isopropyl alcohol, and then acetic acid, rinsed by DI water and blown dry with Ar before use. The Ni plates are heat treated in a CVD quartz chamber at 980°C for 10 mins under the flow of H_2_ gas at a rate of 100 sccm. 20 sccm of CH_4_ was then introduced for 25 mins. The growth was terminated by stopping the flows of H_2_ and CH_4_ gas. The furnace was then turned off and the samples are cool down to room temperature in Ar ambient (300 sccm). These graphene samples were then examined by a confocal Raman spectroscopy system. Typical optical images of these graphene on Ni plates and the corresponding Raman spectra are shown in Figure S5. We then transfer the as-grown graphene from the Ni plate to oxidized Si substrates using thermal release tape (Semiconductor equipment Corp., #3195 MS). These graphene/Si samples are used for the subsequence growth of CVD BNNTs. As suggested by Raman spectroscopy and SEM imaging in Figure
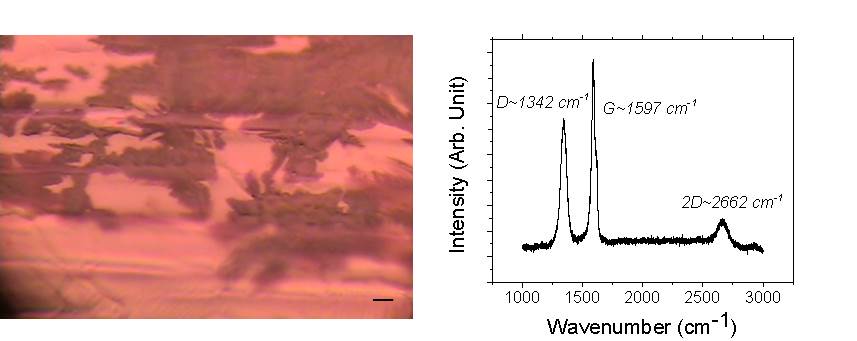
S6, we conclude that BNNTs could not be grown on these CVD graphene.

Figure S5: Optical Images of graphene grown on a Ni plate (left, scale bar = 2 μm) and the corresponding Raman spectra (right). As suggested by the Raman spectra, these are multilayered graphene.


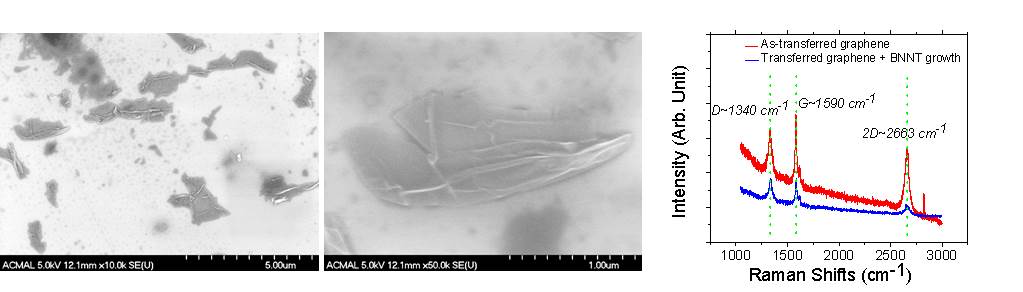


Figure S6: SEM images of the transferred graphene after BNNT growth. Raman spectra of the transferred graphene before and after the growth of BNNTs. No BNNTs are detected.

1. **Characterization by four-probe scanning tunneling microscopy (4-probe STM)**

All graphene-BNNT heterojunctions were characterized at room temperature using a 4-probe STM system under ultrahigh vacuum (base pressure <2×10^10^ Torr) at Oak Ridge National Laboratory’s Center for Nanophase Materials Sciences. This system allows STM measurements and scanning electron microscopy (SEM) imaging. The as-grown graphene-BNNT heterojunctions on oxidized Si substrates (with 500 nm thermal oxide) were mounted on a sample stage, where gate potential can be applied in a back gate configuration. Electrochemically etched tungsten tips are used as the STM probes. After identifying an isolated heterojunction under SEM imaging, one tungsten probe was used to establish electrical contact on the graphene sheet with the guidance of the STM scanner. Another probe was directly contacted on the BNNT at a desired distance, *d* from the heterojunction. Potential differences were then applied across the two probes to characterize the room temperature transport properties across the graphene-BNNT heterojunctions. Figure S7 shows the linear current-voltage (*I-V*) characteristic of the graphene without contacting the BNNTs. This linear *I-V* behavior is very different from the switching characters across the graphene-BNNT junctions.

The STM system allows us to precisely control the Z position of the probes at an atomic resolution. This allows us to land our probes on graphene and BNNT surfaces in a controlled and reliable manner. For measurements on a heterojunction, we first make sure that Ohmic contacts can be obtained on the graphene as shown in Figure S7. Then, we keep one probe on a same spot of graphene, and use another probe to touch the BNNT at a minimum load. Pressing the BNNT harder can be easily observed under SEM as this will move the BNNT. Under excessive force, one can even see the bending of the BNNT and therefore can be easily avoided in our experiments. In principle, our approach can eliminate the effect of contact resistance.

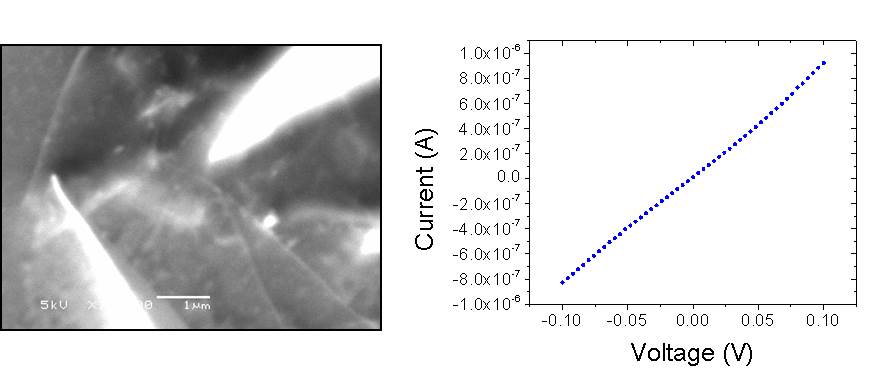


Figure S7. Two STM probes in contact with the graphene surface and the corresponding linear current-voltage (*I-V*) plot.

For the measurement on a vertical graphene-BNNT heterojunction, the STM probe was first approached near the surface of the sample and then lifted up until a contact with the freestanding BNNT was established. By monitoring the current noise level, we can tell when the STM probe is touching the vertically aligned BNNT as indicated by a sudden increase of the current noise level. By this way, we are sure that the STM probe is contact with the protruding BNNT and therefore confirm that it is a vertically aligned BNNT. The full data set of *I-V* characteristic across a vertical graphene-BNNT heterojunction at various distances *d* is shown in Figure S8a to S8e. As shown in Figure S8f and S8g, very low current are detected at *d* = 0.94 μm and 1.23 μm. *I-V* for *d* = 0.62 μm and 0.40 μm are quite identical. Much higher current are detected at *d* = 0.10 μm, and the *I-V* curve is very reproducible.

We further extracted data from Figure S8g, such that the dependent of current versus distance *d* can be evaluated. As shown in Figure 2d, current flows across the heterrojunction is exponentially decrease with the increase of distance *d*, as consistent to a graph plotted in a logarithmic scale (Figure 2f). This result suggests that current is not a simple linear dependent of distance *d*., i.e., not due to the resistance of the BNNT.

The gate effect on the graphene-BNNT heterojunction was evaluated. As shown in Figure S8h, the potential from the back gate is shielded by the metallic graphene sheet.


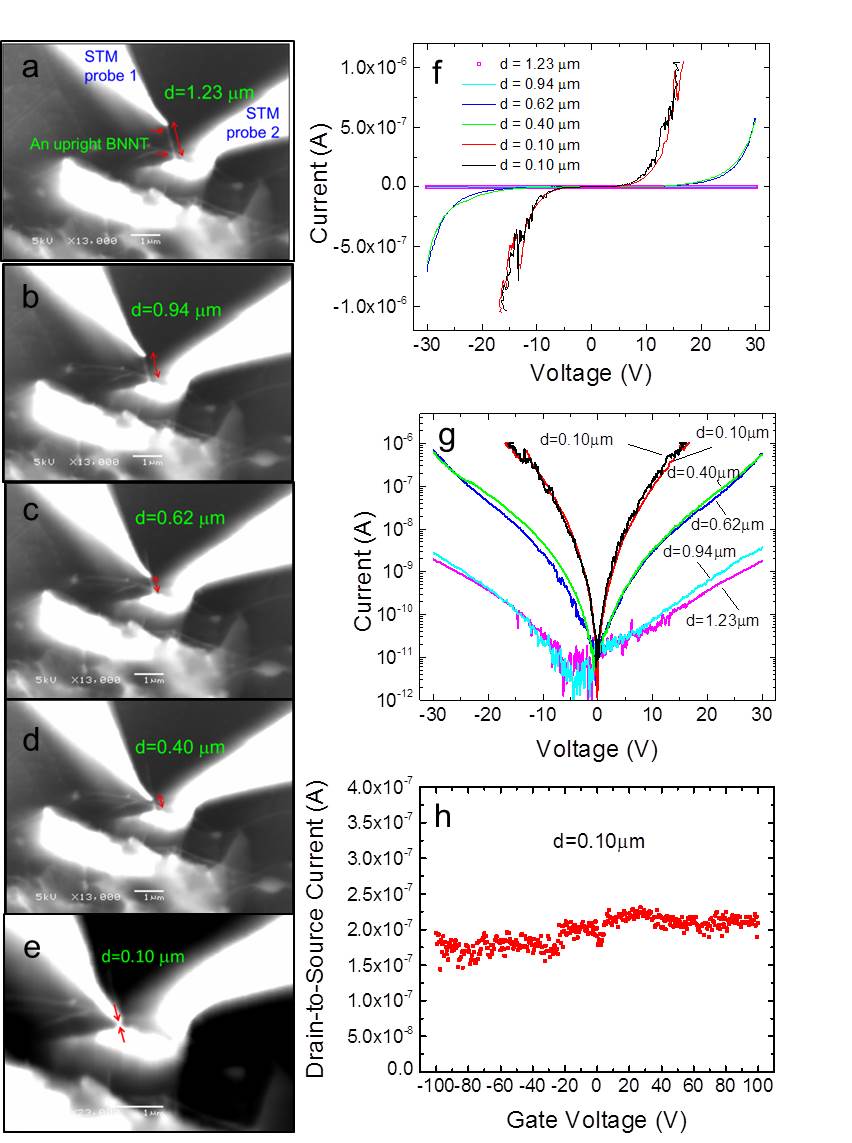


Figure S8: The full data set for *I-V* characteristic across a vertical grephene-BNNT heterojunction.

1. **Theory**

Electronic structure calculations based on Density Functional Theory (DFT) were performed using the SIESTA code. We used the Gradient Generalized Approximation (PBE-GGA) [^6^](#_ENREF_6) for the exchange-correlation functional. The valence electrons of Kohn-Sham wave-functions were expanded with double-ζ polarized (DZP) basis set. The norm-conserving pseudo-potentials [^7^](#_ENREF_7) were used for the core electrons. For the geometry optimization, we used a grid of (12x12x1) k-points and the structures were fully relaxed with residual forces in each component of the atoms smaller than 0.01 eV/Å. DOS was calculated using the grid of (100x100x1) k-points. The non-equilibrium Greens Functions (NEGF) method was used to calculate the current-voltage characteristics using the TranSiesta code. [^8^](#_ENREF_8)

A supercell for graphene was considered with dimensions of 20.24 X 20.24 Å. A BN nanotube with a length of 12 Å was placed perpendicular to the graphene sheet. Firstly, the equilibrium configuration for the pristine graphene and the BN nanotube was obtained. Next, both systems were placed together to form a graphene-BNNT heterojunction. At the BNNT side of the heterojunction, four C atoms were substituted with 2 N and 2 B atoms. The opposite end of the BNNT was passivated with H atoms to avoid the spurious electronic states associated with the dangling bonds. The heterojunction configuration was then optimized, and DOS and electron transport calculations were performed.

The first and second layers of isoplane electron charge distribution at the graphene-BNNT heterojunction are shown in Figure S9. There is a non-negligible charge interaction between graphene and the first layer of BNNT (green color with red/orange localized spots). Electron charges are more localized on the N atoms. For the second isoplane, the uniform red color represents negligible interaction, i.e., there is no mutual influence from the graphene to the second plane of BN atoms of the BNNT. Apparently, DOS of the graphene have been modified at by the heterojunction but not on the BNNTs.


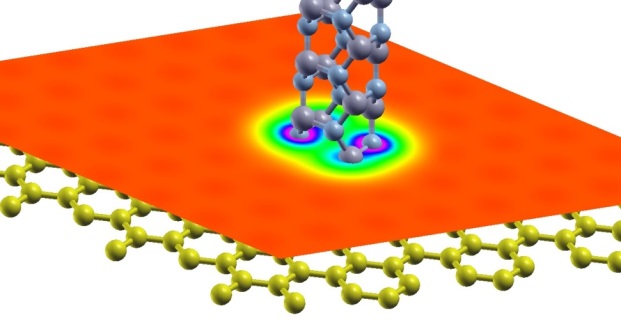

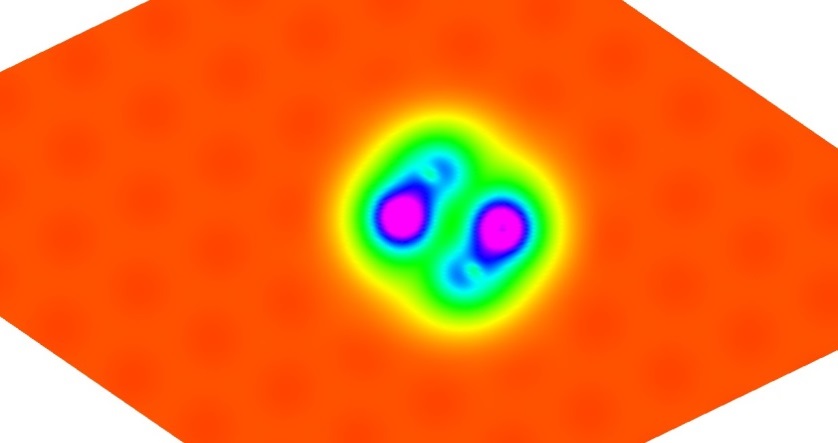

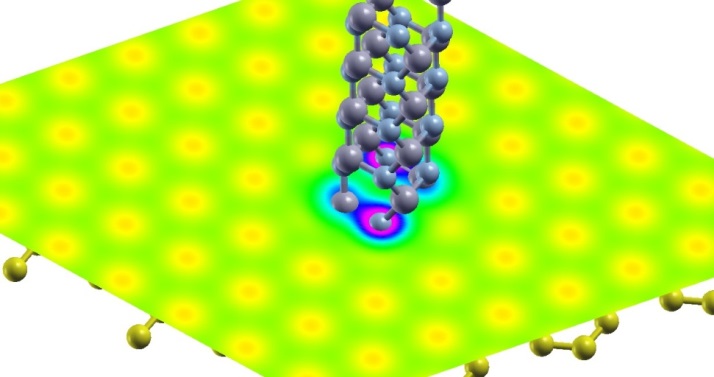

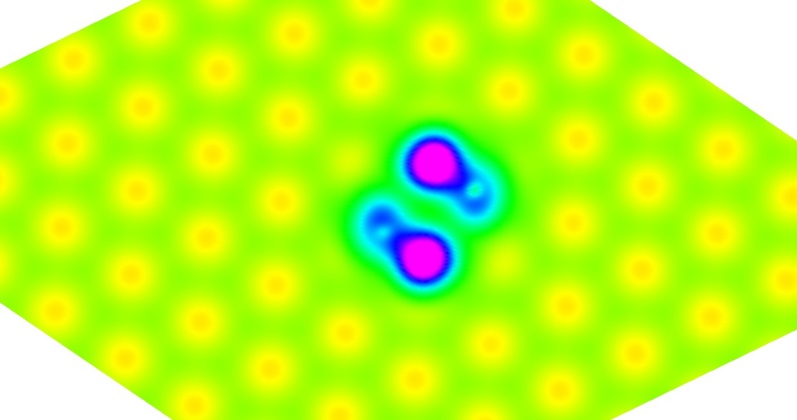


B

N

B

N

B

N

B

N

Figure S9. The first (green) and second (red) layer of isoplane electron charge distribution at the graphene-BNNT heterojunctions

**References**

1 Mohiuddin, T. M. G. *et al.* Uniaxial strain in graphene by Raman spectroscopy: G peak splitting, Gruneisen parameters, and sample orientation. *Physical Review B* **79**, 205433 (2009).

2 Ferrari, A. C. & Basko, D. M. Raman spectroscopy as a versatile tool for studying the properties of graphene. *Nat Nanotechnol* **8**, 235-246 (2013).

3 Calizo, I., Bejenari, I., Rahman, M., Liu, G. & Balandin, A. A. Ultraviolet Raman microscopy of single and multilayer graphene. *J Appl Phys* **106**, 043509 (2009).

4 Hohenberg, P. & Kohn, W. Inhomogeneous Electron Gas. *Physical Review* **136**, B864-B871 (1964).

5 Kohn, W. & Sham, L. J. Self-Consistent Equations Including Exchange and Correlation Effects. *Physical Review* **140**, A1133-A1138 (1965).

6 Perdew, J. P., Burke, K. & Ernzerhof, M. Generalized Gradient Approximation Made Simple. *Physical Review Letters* **77**, 3865-3868 (1996).

7 Troullier, N. & Martins, J. L. Efficient pseudopotentials for plane-wave calculations. *Physical Review B* **43**, 1993-2006 (1991).

8 Brandbyge, M., Mozos, J.-L., Ordejón, P., Taylor, J. & Stokbro, K. Density-functional method for nonequilibrium electron transport. *Physical Review B* **65**, 165401 (2002).
